# Numerical analysis of 40 MW HTS motor electromagnetic characteristics for ship electric propulsion

**DOI:** 10.1038/s41598-023-45770-4

**Published:** 2023-11-20

**Authors:** Kai Bo, Junquan Chen, Yapeng Jiang, Dong Wang

**Affiliations:** National Key Laboratory of Science and Technology On Electromagnetic Energy, Naval University of Engineering, Wu-Han, 430033 People’s Republic of China

**Keywords:** Energy science and technology, Engineering, Materials science

## Abstract

In recent years, with the development of ship electric propulsion system, High Temperature Superconducting (HTS) motors have gained attention as a promising potential resolution due to their high-power density and the progression of cryogenic refrigeration technology. We present an electromagnetic characteristics numerical analysis of 40 MW, 120 rpm, HTS synchronous motor which is a semi-superconducting motor: in fact, it has a superconducting rotor composed of YBCO material and a conventional stator. Combining electric resistivity variation of rotor copper shielding sleeve in low temperature environment and YBCO coil critical current, the electromagnetic behavior of the 40 MW HTS synchronous motor is analyzed with non-ferromagnetic stator support structure. To assess the effects of varying stator winding currents and thickness on eddy current losses of the copper shielding sleeve, calculations were done, and a preliminary discussion was initiated. This study has certain reference significance for electromagnetic design and optimization of multi-ten MW HTS motors, especially used in ship electric propulsion system.

## Introduction

The International Maritime Organization's requirements for ship emissions are getting stricter, especially in the field of environmental control. Compared with mechanical propulsion system, electric propulsion system can reduce fuel consumption, and even achieve low pollution, which is the first choice for green ecological ships. In addition, the electric propulsion system has good low-speed propulsion characteristics and fast start-stop characteristics, flexible operation, high efficiency, low vibration noise, easy installation, and layout. Therefore, it is suitable for military ships, ships with high mobility requirements and ships with special working properties^1^. The progress made in the field of power electronics, motors, energy storage and control technology, have given new boost to the electric propulsion sector for naval applications. Consequently, with continuous development in electric propulsion, especially the integrated power system for ships, the requirement of higher power density and efficiency of propulsion motor has lie in the future application.

As well known, the power density is proportional to the air gap magnetic flux density and the stator current. Although the currently rare earth permanent magnets have a magnetization residual of 1.3 T, the coils made using 2G YBCO or 1G BSCCO tapes can produce magnetic flux densities up to 14 T. Therefore, using the superconducting materials, the power density can be increased by 5 to 10 times. HTS motor is an important potential technology to obtain the goal of higher power density and smaller volume.

There already exist many papers on modeling and testing of HTS generators and motors^2^. In particular, much research are focused on DC driving multi-MW rated prototype for wind-generation application^3–8^. Ref^5^, for example, proposed a dual stator HTS wind generator with stationary seal according to the air-gap magnetic field modulation theory. A 2 MW, 20 rpm superconducting direct drive wind turbine has been successfully developed and described in Ref^6^: the superconducting coil cooling mode adopted allow a precooling time of about 3 days, which is much faster than that of the superconducting direct drive motor designed by Eco Swing project (about 14 days). However, there is little information and only few concepts about multi-MW class HTS motor for ship electric propulsion. Such as Ref^2^ reported factory tests on a 36.5 MW HTS propulsion motor, whereas Ref^7^ demonstrated an HTS 1 MW, 500 rpm propulsion motor having HTS coils made with BSCCO and placed on the four pole pairs rotor.

This paper focuses on a numerical analysis of a 40 MW synchronous motor in radial flux configuration for ship electric propulsion. The considered electrical machine has a conventional winding placed on the stator and a superconducting winding placed on the rotor. The rotor configuration is salient pole and will operate at cryogenic temperatures, whereas the stator will operate at temperature around 25 °C. The goal of this study is investigating the electromagnetic field distribution and the eddy current losses on the rotor copper shielding sleeve. Based on the Maxwell equations, a simplified 2-D electro-magnetic model is established to determine the critical current of each HTS coil. The obtained simulation and experimental results are also compared.

For HTS rotating motors, the rotor copper shielding sleeve is a vital component in safeguarding the rotor HTS coil from the negative harmful effects of high-order harmonics, due to AC windings on stator, as well as delaying the rising speed of the stator short-circuit pulse magnetic field at the rotor superconducting winding. In this case, low resistivity and thick wall shielding layers are preferred. If the HTS field winding needs to respond quickly to reactive power compensation, the rotor copper shielding sleeve will reduce the slope of forced excitation; so, high resistivity and thin wall are required. Because these two characteristics are completely contradictory, a correct trade-off must be found. In addition, the rotor copper shielding sleeve also serves as a vacuum chamber to maintain a low-temperature environment and must have sufficient mechanical strength to prevent unexpected deformation caused by centrifugal stress during high-speed rotation. The induced eddy current on the rotor copper shielding sleeve by AC magnetic field due to the armature current, reduce the shielding effect and causes unnecessary heat loss.

To this aim, the influence of stator armature current on eddy current loss of rotor shielding sleeve are calculated. Better understanding of the magnetic flux density distribution and eddy current sure enough, is of significant value for the design of the electromagnetic and the thermal system. Therefore, setting the electric resistivity value of the rotor copper shield sleeve at cryogenic temperature environment, the electromagnetic behavior such as magnetic flux density, torque and core loss are analyzed in detail.

## Electromagnetic model

### Hypothesis

**Figure 1:**
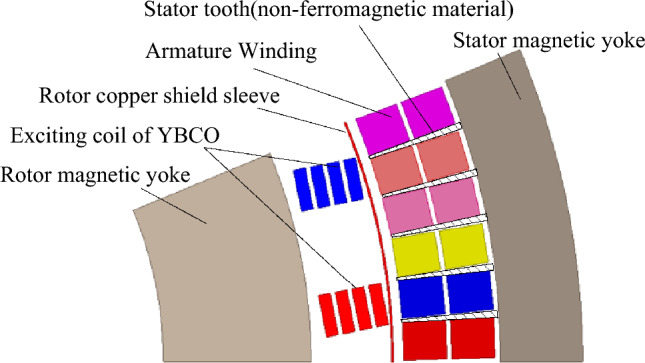
Schematic diagram model of 40 MW HTS synchronous motor (1/16).

Given the complex structure and symmetry of the considered motor, a 2D electromagnetic model, corresponding only to a 1/16 of the machine circumference sector, is implemented in ANSYS Electronics Desktop 2020 R2 environmental (see Fig. 1). Table 1 shows the key parameters of the 40 MW HTS motor analyzed. The resistivity of rotor copper shield sleeve at the cryogenic temperature is considered for its eddy current loss calculating. To reduce the computing time, the effects of mechanical properties varying, especially stress and deformation caused by the inconsistent shrinkage at cryogenic temperature as well as the effect of displacement current and cooling systems, are not considered.

**Table 1:** Key parameters.

Parameters	Values
Poles number	16
Slot number	96
Phases number	6
External diameter of stator magnetic yoke/mm	3300
Internal diameter of stator magnetic yoke/mm	2970
Axial length/mm	800
Shielding sleeve thickness /mm	4
Rated speed/rpm	120

### Parameters

Table 1 shows the key parameters of the 40 MW HTS synchronous motor.

### Maxwell equations

The electromagnetic field are described as Ref^4^. According to Maxwell’s equations, we have:

1 $$ \nabla \times \overrightarrow {E} = - \frac{{\partial \overrightarrow {B} }}{\partial t} $$

2 $$ \nabla \times \overrightarrow {H} = \overrightarrow {J} $$

where *E* is the magnitude of electric field intensity, *B* is the magnetic flux density, *H* is the magnetic field intensity, and *J* is the current density. From constitutive law, it is obtained that:

3 $$ \overrightarrow {B} = \mu_{0} \mu_{R} \overrightarrow {H} $$

where *μ*_*0*_ is the free space permeability, and *μ*_*R*_ is the relative permeability of the studied material. According to Ohm’s law, it is obtained that

4 $$ \overrightarrow {J} = \sigma \overrightarrow {E} $$

where *σ* is the conductivity of the material. Based on the previous (1), (2), (3), the general form H-formulation can be obtained to solve the electromagnetic equations in ANSYS environmental:

5 $$ \frac{1}{\sigma }\nabla \times (\nabla \times \overrightarrow {H} ) + \mu_{0} \mu_{R} \frac{{\overrightarrow {\partial H} }}{\partial t} = 0 $$

**Figure 2:**
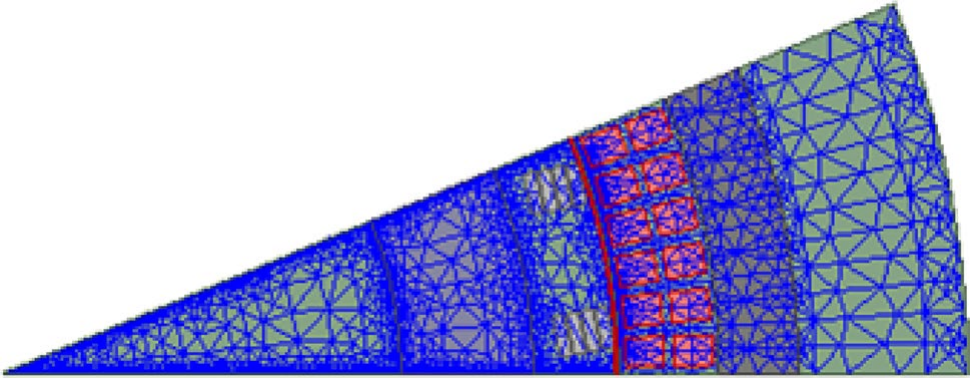
Schematic diagram of numerical model mesh (1/16 model).

The mesh of the machine sector analyzed is shown in Fig. 2, whereas the material properties of the HTS coil will be described in the following.

### Resistivity at cryogenic environments

**Figure 3:**
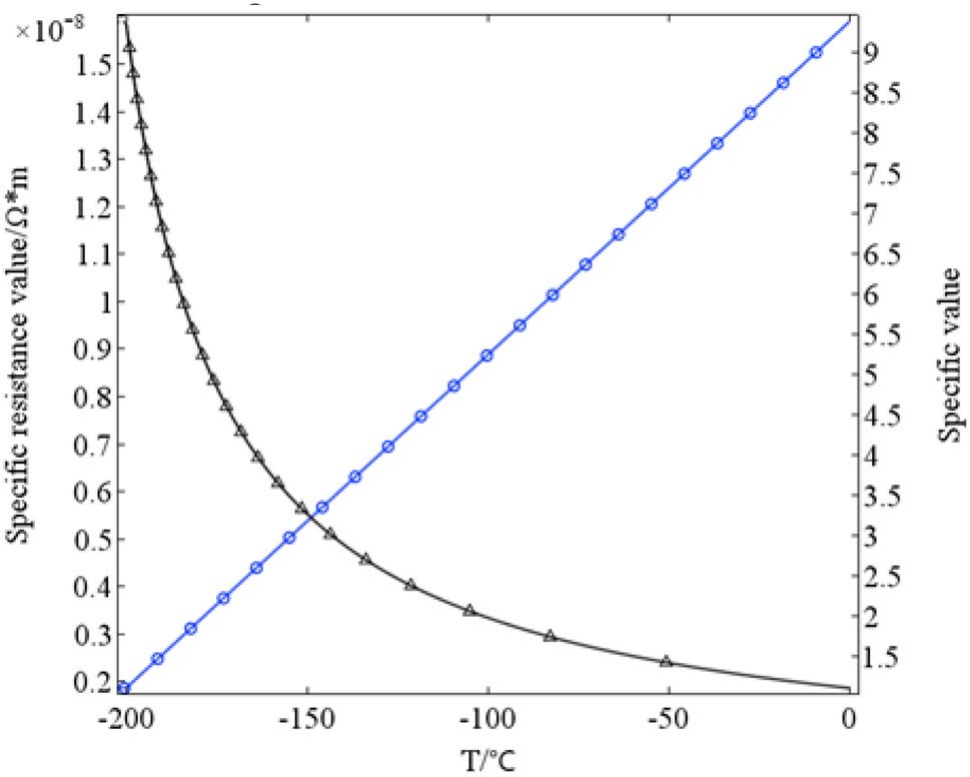
Copper specific resistance and ratio at cryogenic environment.

With the decreasing of temperature (T) of the cryogenic environment, the copper resistivity of rotor shield sleeve decreases. Its value will be different from that of its surroundings and will affect the electromagnetic parameters of the HTS motor. Therefore, it is necessary to quantitatively describe the influence of cryogenic environment on the material properties of rotor shield sleeve. The resistivity of copper decreases with the decreasing of temperature at cryogenic environment. According to Ref.^6^, the resistivity can be fitted to a function related to temperature.

6 $$ \rho_{Cu} (T) = 7.01 \times 10^{ - 11} T + 1.59 \times 10^{ - 8} $$

where *ρ*_*Cu*_ is the resistivity at the temperature T. Equation (6) shows as the resistivity at cryogenic temperature can be calculated and compared with that at room temperature of 25 °C, as shown in Fig. 3.

It can be seen from the fitting data that the resistivity has a linear trend with the temperature increasing. Trend of the specific value ratio of resistivity under different temperature of cryogenic environment and room temperature instead, is not linear with T: the lower the temperature, the more obvious the increment of the specific value ratio.

## Critical current of a HTS coil

### Control equations

As well known, to investigate the HTS tape performance, the magnetic flux density (B) components to be considered are perpendicular and parallel to the tape. For the YBCO tape selected in this paper, the vertical magnetic field is the worst working condition than that of parallel magnetic field, so we concentrate on verifying the working condition of vertical magnetic field. Neglecting the HTS coil end bending part and with reference to Francesco Grilli model^8–11^, a power law E–J (Italic) relationship is used to estimate the critical current density of each HTS coil:

7 $$ \overrightarrow {E} = E_{c} \left( {\frac{{\overrightarrow {J} }}{{J_{c} }}} \right)^{n} $$

Substituting the Eq. (7) in (4), we obtain:

8 $$ \sigma = \frac{{J_{c} }}{{E_{0} }}\left( {\frac{{J_{c} }}{J}} \right)^{n - 1} $$

9 $$ J_{c} (\left| B \right|) = \frac{{J_{c0} B_{0} }}{{\left| B \right| + B_{0} }} $$

where *E*_*c*_ is the electric field strength criterion (1•10^4^ V/m), *E*_*0*_ is the critical electric field strength, *J*_*c0*_ is the critical current density in the self-field, *B* is the external field magnetic flux density, *B*_*0*_ is magnetic flux density in the self-field and n is the resistance transition constant. We used the COMSOL Multiphysics 5.2a software to implement and solve the equations of (7)–(9), and to compute the coil critical current.

### Homogenization process

**Figure 4:**
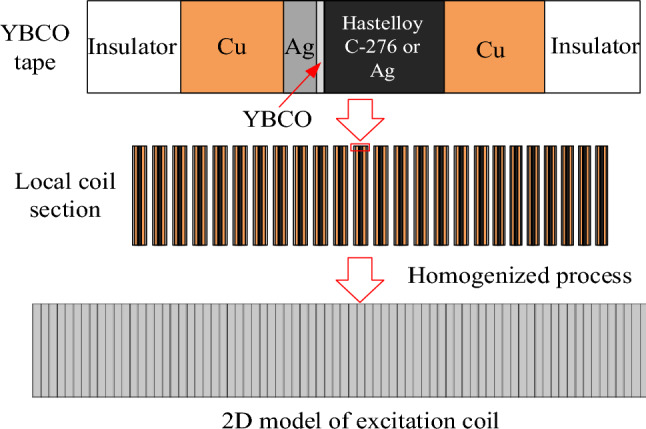
Schematic diagram of homogenization process.

Each HTS coil under investigation is composed of several YBCO tapes, and each YBCO tape contains various layers (YBCO layer, copper layers, silver layer, substrate layers and insulated layers) that are not symmetrical. To reduce numerical modeling intense and time consuming to build a complex model for calculating the critical current of superconducting excitation coil, the homogenization process method^12^ is used to build 2D HTS excitation coil cross section. In this model, a single YBCO tape has been presented as a unit cell composed of all the layers, and only the YBCO material’s volume fraction has been considered, as shown in Fig. 4.

**Figure 5:**
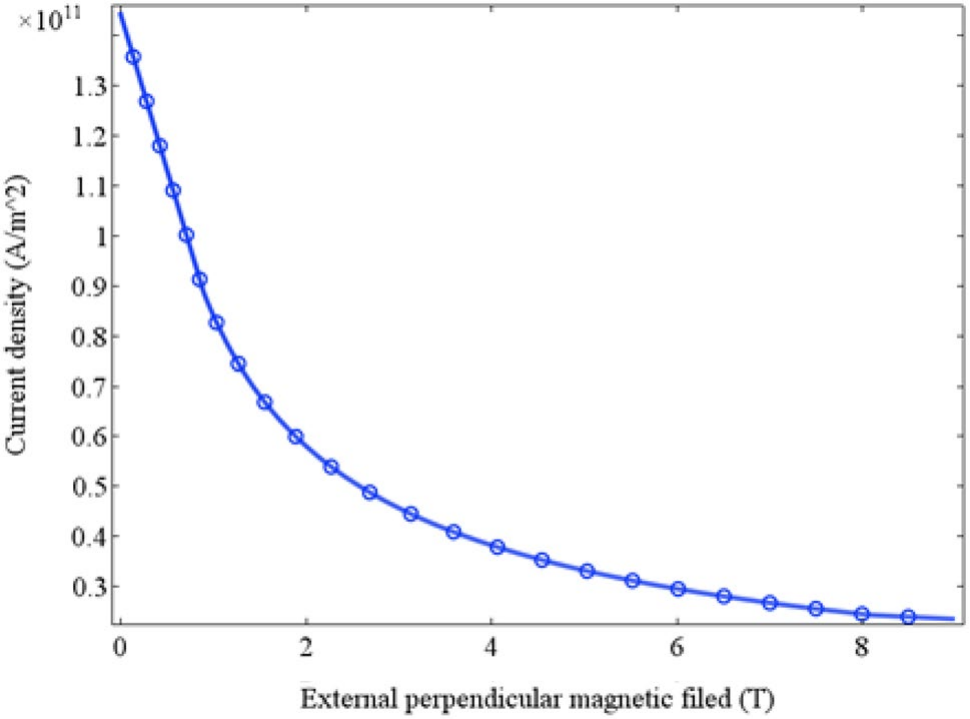
Dependency of critical current density and perpendicular magnetic induction intensity.

After homogenization process, the critical current density of HTS coil also need to be converted accordingly and calculated as engineering current of HTS tapes. The critical current density will be affected by the vertical magnetic induction. The relationship between the critical current density and vertical magnetic induction intensity (provided by Shanghai Superconducting Technology Co., Ltd.) it can be fitted as shown in Fig. 5. The 4.8 mm red copper armored YBCO superconducting tape, selected in this paper, has a thickness of 180 μm and a critical current of 240 A at 30 K, when the vertical magnetic field is 4 T.

### Experimental measurements

**Figure 6:**
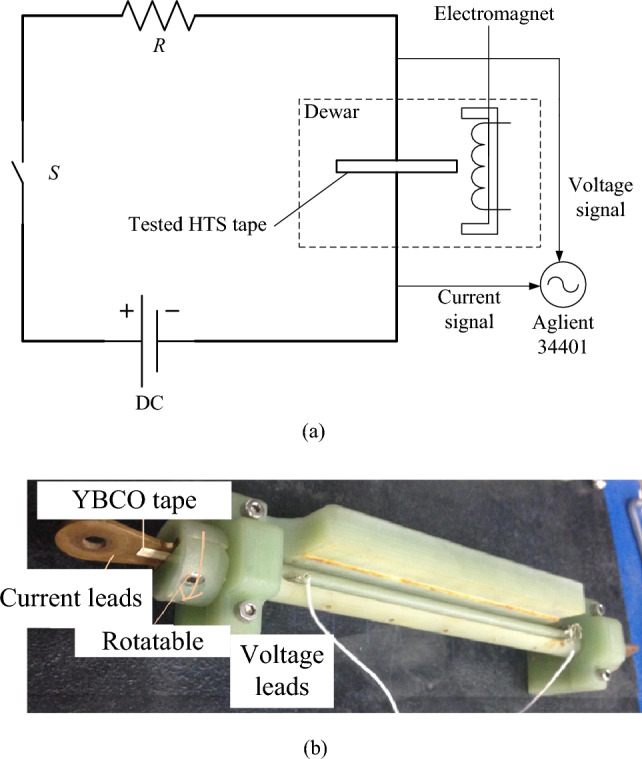
Schematic diagram of experiments. (**a**) Experimental circuit (**b**) Angle clamping apparatus for YBCO tape sample.

To verify the effectiveness of the critical current model, an experimental apparatus was set-up in our labs (Fig. 6a,b). To this aim, we designed the electromagnet, HTS tape fixed device, and measurement program through MATLAB 2018b, which can automatically collect and processing data.

To measure the voltage across of HTS tape, an Agilent 34,401 has been selected and connected to a computer through a GPIB-488 interface, which can measure a minimum of 0.1 μV. A FLUKE 8808A digital multimeter is used to measure the voltage of the protection resistor, R; dividing this voltage value by the resistance value of the protection resistor, the main circuit current value is obtained and acquired. In order to provide a background magnetic field for the HTS tape under test, we need to place an electromagnet in an open Dewar bottle. Therefore, an electromagnet with a maximum magnetic flux density of 0.5 T, a designed power frequency of 50–400 Hz, an air gap of 15 mm, and a length of 140 mm has been designed. The electromagnet is wound with 375 coils on both sides, and a current of about 15 A is required to reach the maximum magnetic field value when the HTS tape is inserted into the air gap of the electromagnet.

The adjustment of parallel and vertical magnetic fields can be achieved using special equipment. Figure 6b shows, the experimental fixture designed for the HTS tape. It is composed of two insulation plates inside which 16 cm of the HTS tape and a copper electrode have been inserted. The superconducting strip plate and copper electrode are fixed with screws, and after compression, it can avoid force movement when the strip is energized. The voltage leads are then welded to the HTS tape and pressed tightly with screws. Ensuring good contact at the welding points means reducing the contact resistance and the consequent generation of heat.

**Figure 7:**
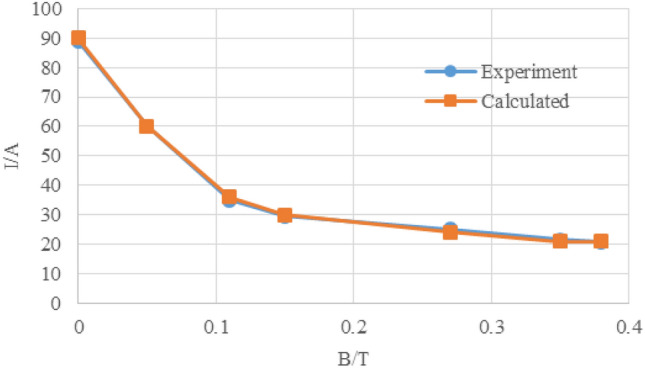
Critical current compared between calculated and experimental value.

In the vertical magnetic field experiments, we can get the result by analyzing the experimental results, HTS tapes more easily lose superconductivity in the AC magnetic field than in the DC magnetic field. Under the action of 0.35 T DC external magnetic field, YBCO strip still has 25% critical current under self-field. The critical current of YBCO strip under DC magnetic field will also be greatly reduced, but the reduction speed is slow, and it can still maintain a high critical current under the action of large DC magnetic field. The validity of the calculated HTS tape critical current based on the Eqs. (7)–(9) is verified by comparing the experimental results with the calculated values, as shown in Fig. 7.

## Results

### Critical current distribution of HTS coil

**Figure 8:**
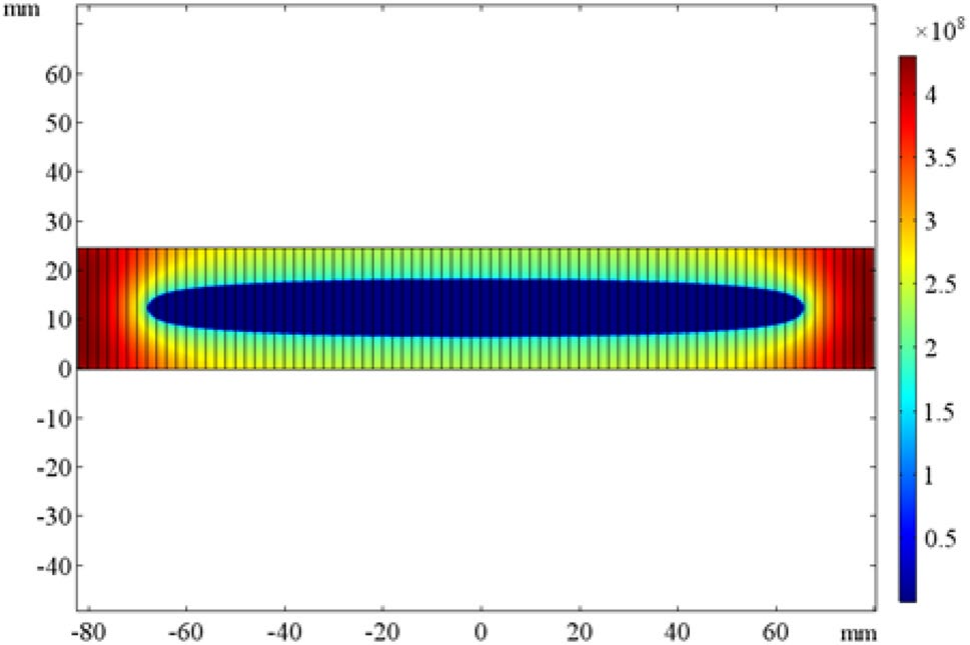
Current density distribution of the HTS coil cross-section.

The critical current density distribution of the HTS coil cross-section is shown in Fig. 8. As can be seen, the part near the inter-tapes is small and, at the same time, it can be found that there are differences among multi-turns. The areas with lager critical current density at the innermost and outermost turns are more, while the areas with larger critical current density at the intermediate turns are less. The critical current distribution of the HTS coil presents a concave curve with low middle and high sides.

This means that, moving from inside turns to outside turns, the critical current value increases from 169 to 225 A at 30 K, while 30 K is one of the YBCO rated working condition. Therefore, when the coil current will approach the critical value, each turn in the middle of the HTS coil will first face the danger of quench. According to the obtained results and to ensure the safety of the HTS coil operating, a 40% approximately margin is generally reserved on the coil current value; therefore, the overall current of the HTS coil is set to 135 A, and the turns number is set to 1694.

### Characteristics of electromagnetic field

**Figure 9:**
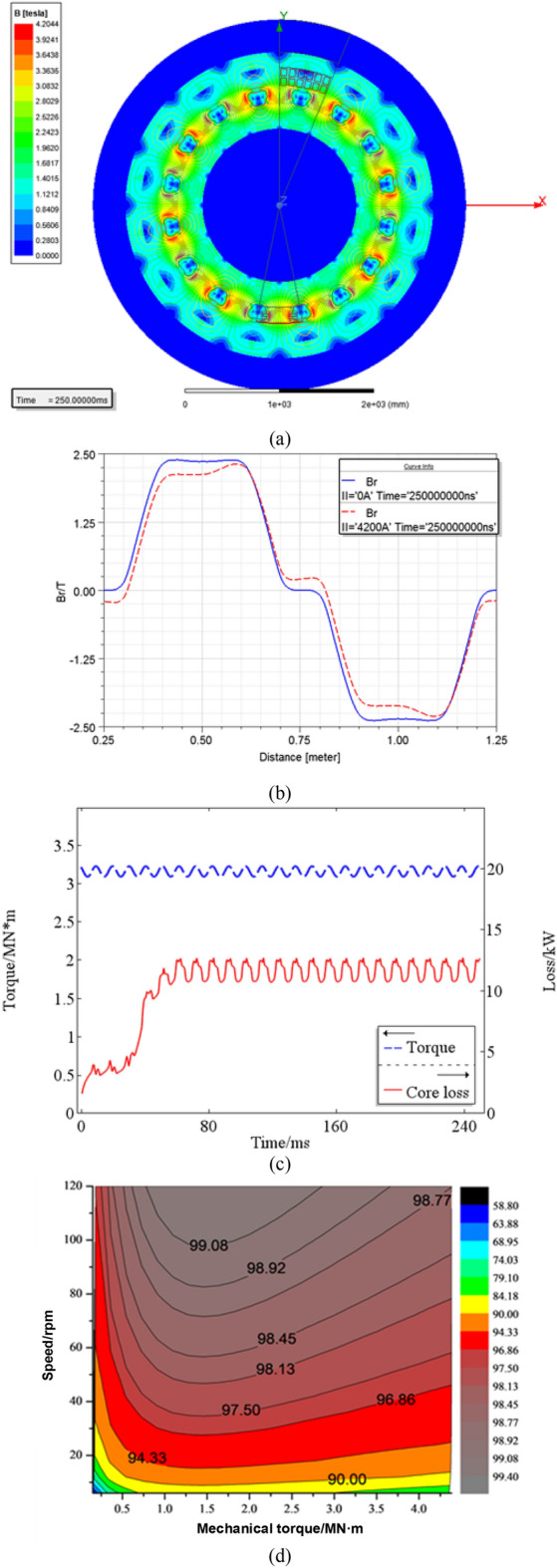
Magnetic characteristics of the HTS motor, they should be listed as: (**a**) Magnetic flux density distribution; (**b**) Air-gap magnetic flux density ;(**c**) Time evolution of calculated torque and core loss;(**d**) MAP figure.

With reference to the Fig. 1, the stator yoke and the rotor yoke provide a closing path to the magnetic flux lines in the radial direction when they are made of ferromagnetic material. This is beneficial to reduce the magnetic flux leakage. The ANSYS simulation results, reported in Fig. 9a, show that both are saturated when the stator current is 4200 A; in particular, the stator support structure made by silicon steel of 35WW270 is more prone to magnetic saturation than the non-ferromagnetic as G10 material. Due to the induced eddy current on the shielding sleeve of the rotor, the influence of the stator AC magnetic field on the superconducting coil is reduced but undesired heat loss can be observed.

According to the simulation results of the Fig. 9a, the air-gap magnetic flux density under no-load and rated load are shown in Fig. 9b: the air gap magnetic density amplitude is about 2.23 T. With reference to Fig. 9c: the eddy current loss of sleeve is 12.69 kW; the core loss is 6.63 kW; the torque is 3.16 MN•m. The HTS motor MAP is shown in Fig. 9d. As can be seen, the entire motor has a large high efficiency area with a value over 90% in operating conditions and exceeding 95% within the torque range of 5 to 100%.

## Discussions

### Influence of winding current on eddy current and core loss

**Figure 10:**
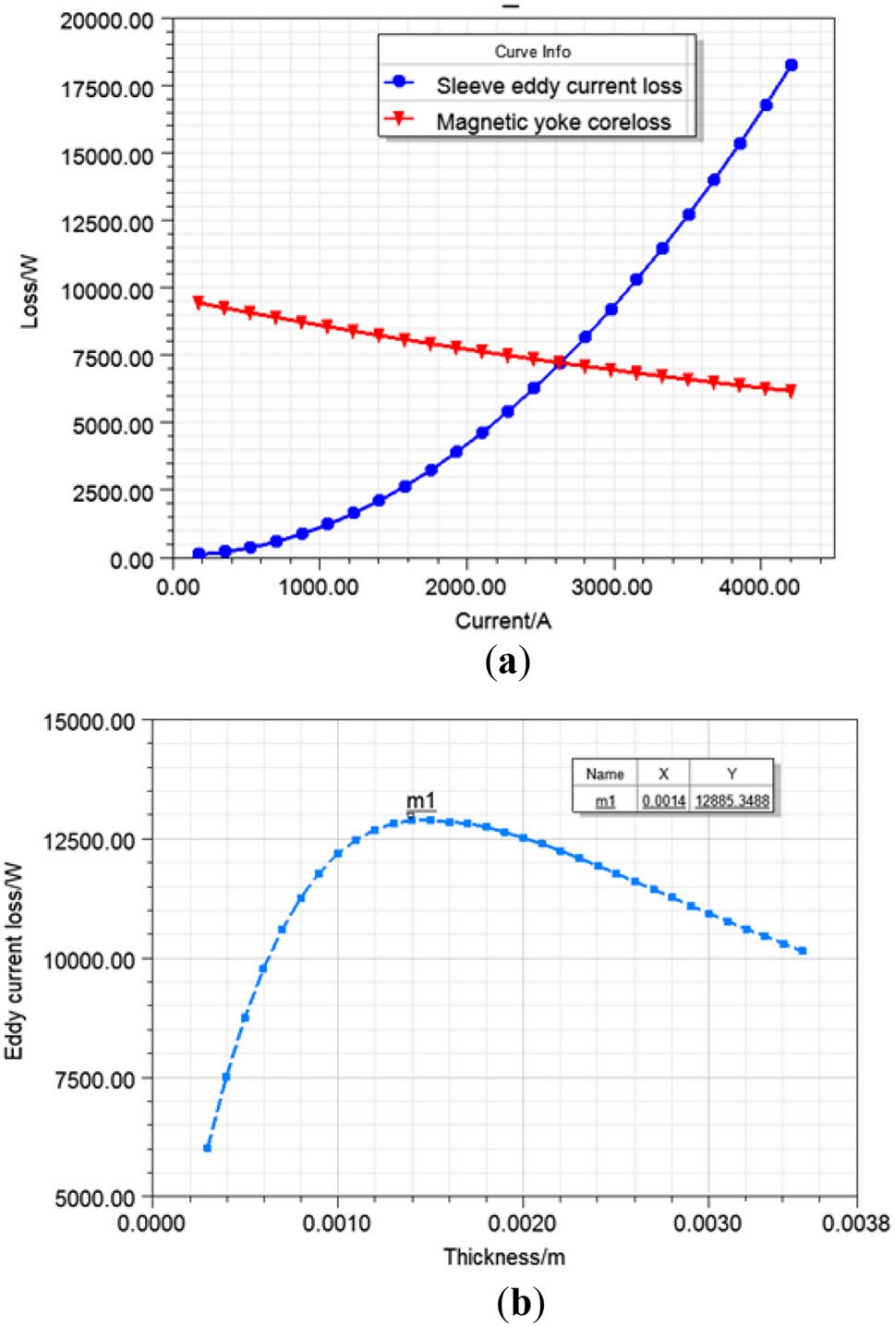
Numerical results (**a**) The eddy current loss of sleeve shield and motor core loss under different stator current; (**b**) The average eddy current loss of sleeve shield at its different thickness.

With the increase of the stator winding current, the eddy current loss of the copper shielding sleeve also increases accordingly, while the magnetic yoke core loss including rotor and stator is inversely proportional to the stator current. The magnetic yoke core loss trend is decreasing along with the stator current, but overall change is not large as shown in Fig. 10a.

### Influence of sleeve shield thickness on eddy current loss

Figure 10b shows the relationship between the thickness of the rotor copper shielding sleeve and the average value of eddy current loss. With the increase of thickness, its eddy current loss increases up to the maximum value of 12.89 kW in correspondence of 1.4 mm. Then, for thickness value greater than 1.4 mm, the average eddy loss decreases.

It was demonstrated through this result that the thickness of the rotor copper shielding sleeve had not been chosen too thickly in order to abate the rotor weight and financial outlay. We must be particularly mindful of the eddy current loss maximum critical value of rotor copper shielding sleeve to prevent any chance of a heightened stress on the refrigeration and heat dissipation system.

## Conclusions

This paper presents an electromagnetic analysis of 40 MW HTS motor. Considering the electric resistivity variation of rotor copper shielding sleeve at low temperature, the influence of different stator currents on eddy current losses of the rotor copper shielding sleeve and magnetic yoke core loss is analyzed. The eddy current loss of rotor copper shielding sleeve is calculated under different thickness. This study has certain reference significance for electromagnetic design and optimization of multi-ten MW HTS motors, especially used in ship electric propulsion system.

## Data Availability

All data generated or analysed during this study are included in this published article.
